# Dietary diversity of formal and informal residents in Johannesburg, South Africa

**DOI:** 10.1186/1471-2458-13-911

**Published:** 2013-10-02

**Authors:** Scott Drimie, Mieke Faber, Jo Vearey, Lorena Nunez

**Affiliations:** 1 Human Nutrition, Interdisciplinary Health Sciences, Stellenbosch University, Francie van Zijl Avenue, Tygerberg 7505, South Africa; 2 Nutritional Intervention Research Unit, Medical Research Council, PO Box 19070, Tygerberg 7505, South Africa; 3 African Centre for Migration and Society, University of the Witwatersrand, PO Box 76, Wits, Johannesburg 2050, South Africa

**Keywords:** Dietary diversity score, Nutrition security, Informal settlements, Johannesburg

## Abstract

**Background:**

This paper considers the question of dietary diversity as a proxy for nutrition insecurity in communities living in the inner city and the urban informal periphery in Johannesburg. It argues that the issue of nutrition insecurity demands urgent and immediate attention by policy makers.

**Methods:**

A cross-sectional survey was undertaken for households from urban informal (n = 195) and urban formal (n = 292) areas in Johannesburg, South Africa. Foods consumed by the respondents the previous day were used to calculate a Dietary Diversity Score; a score < 4 was considered low.

**Results:**

Statistical comparisons of means between groups revealed that respondents from informal settlements consumed mostly cereals and meat/poultry/fish, while respondents in formal settlements consumed a more varied diet. Significantly more respondents living in informal settlements consumed a diet of low diversity (68.1%) versus those in formal settlements (15.4%). When grouped in quintiles, two-thirds of respondents from informal settlements fell in the lowest two, versus 15.4% living in formal settlements. Households who experienced periods of food shortages during the previous 12 months had a lower mean DDS than those from food secure households (4.00 ± 1.6 versus 4.36 ± 1.7; p = 0.026).

**Conclusions:**

Respondents in the informal settlements were more nutritionally vulnerable. Achieving nutrition security requires policies, strategies and plans to include specific nutrition considerations.

## Background

Undernutrition in developing countries has been called the 'silent emergency’, which has recently gained attention from international donors and national policymakers [1]. Yet, political debates around nutrition insecurity in the African context have scarcely recognised the urban dimension facing the continent [2,3]. Urban planners and policymakers prioritise issues concerning unemployment, overcrowding, decaying infrastructure and declining services, as these remain the more visible dimensions of the development needs of cities. This reflects a poor understanding of the critical role of nutrition for health and development and its potential role to lift African cities out of a spiral of poverty [4]. Indeed, the nutrition transition underpinned by dietary changes in the urban context and associated challenges posed by undernutrition has occurred in the context of massive rural–urban migration and rapid urbanization across the continent [5,6]. This poses a major threat to public health with impacts on the poor – and therefore the most food insecure – being the most damaging [6].

Food insecurity is defined as “the lack of physical, social and economic access to sufficient, safe and nutritious food that meets the dietary needs and food preferences for an active and healthy life” [7]. The economic access to food that is safe and nutritious should resonate with urban development planners and practitioners particularly as urbanization intensifies on the African continent. In the early 1990s, two-thirds of all Africans lived in rural areas with future estimates that around 2030, Africa will enter its urban age with 759.4 million people - half of its total population - living in cities [8]. In terms of sub-regions, Southern Africa has the highest rate of urbanization in the world and is expected to be two-thirds urbanized by 2050 [8].

One result of this rapid urbanization is that food insecurity, particularly access to safe and nutritious food, will increasingly be an urban problem [3]. People’s food security is heavily tied to market forces, which in turn are prejudiced by socio-economic conditions that limit their ability to access food, largely through food purchase (though food may also be obtained through exchange or gifts) [9]. This is particularly accentuated in urban areas. Garrett and Ruel found the percentage of the population to be found energy deficient in terms of food consumption was higher in urban areas in most of ten countries that were investigated in sub-Saharan Africa [10]. This correlated with research on the urban face of food and nutrition security, emphasising health and food security as important prerequisites for nutrition security, which highlighted the magnitude of rural–urban and intra-urban health differences in mortality, morbidity, and malnutrition [11,12].

The causes of nutrition insecurity in urban areas are exacerbated by issues related to urban living such as a greater dependence on cash income; weaker informal safety nets; greater labour force participation of women and its consequences for child care; lifestyle changes, particularly diet and exercise patterns; greater availability of public services, but questionable access by the poor; and greater exposure to environmental contamination [12]. Also, urbanization is associated with a number of unhealthy dietary changes such as increased consumption of saturated and trans fats, sugars, salt and processed foods. These dietary changes are occurring at a rapid rate in developing countries and at earlier stages of economic and social development, and as a result the global burden of obesity and other non-communicable diseases is shifting towards the poor [13]. Dietary quality in particular has therefore become a very important health issue in the context of rapid urbanization. South Africa is in the non-communicable diseases phase of the nutrition transition [14], with the urban poor being disproportionally affected [15]. South Africa has adequate food supply at the national level [16], yet a substantial proportion of households are at risk of hunger or are experiencing hunger [17].

With the shifts in demographics, human health factors have a powerful impact on food systems through mechanisms such as migration, shifts in labor force and demographic structure. In turn these factors can lead to poor nutrition and food insecurity. A variety of foods in the diet is needed to ensure an adequate intake of essential nutrients. Dietary diversity can be used as a proxy measure of the nutritional quality of the diet and for the access dimension of household food security (18). A low dietary diversity is associated with stunted growth in children [18,19], and a higher probability of metabolic syndrome [20] and cardiovascular risk factors [21] in adults. The South African population in general consumes a diet with little variety [22] and is therefore nutritionally vulnerable.

This paper considers the question of nutrition insecurity and dietary diversity in communities living in the inner city and the urban informal periphery in Johannesburg, the wealthiest and most populous of South African cities. It considers in particular those residing informally and those residing formally. The term 'informal settlement’ is used to describe unplanned settlements that involve people claiming land and constructing their own housing without legal tenure. As a result, many informal settlements are poorly located and inadequately serviced, experiencing multiple challenges in accessing basic services such as water, sanitation and refuse collection. The aim of this study was to determine the dietary diversity for these two groups.

## Methods

### Study population and design

This paper draws on key findings from the Johannesburg case study of the Regional Network on AIDS, Livelihoods and Food Security (RENEWAL) research project that set out to explore the linkages between HIV, migration and urban food security [23,24]. A multidisciplinary advisory group guided the study, with ethics approval obtained from the University of the Witwatersrand Medical Research Ethics Committee (protocol number M071125). A cross-sectional household survey was undertaken in 2008 designed to gather information on all members of the household and obtaining data from 487 households. A range of data was collected including migration histories; household composition; access to legal, social and health services; livelihood choices; social networks and linkages; food security, and interlinked health and development indicators. Respondents were either the head of the household, or another adult household member able to provide information on all members of the household.

In order to explore intra-urban inequalities and the interlinked deprivations encompassing urban poverty, the survey sample was divided between one purposively selected peripheral urban, informal settlement and an inner-city area made up of three purposively selected suburbs in the dense inner city. The informal settlement included in this study was selected as it represents the complexity of peripheral, informal urban space that is currently being upgraded by local government. Located on a former mining compound, the diverse housing types include self-constructed shacks, former mine worker accommodation, recently constructed RDP (the South African government’s Reconstruction and Development Programme) housing, and “transit” housing (shacks constructed by local government to house residents who’s shacks have been removed by local government and are awaiting RDP housing). The three central-city suburbs were purposively selected from the inner city as areas where cross-border migrants are known to reside [25]. These suburbs represent planned, high-density residential areas that were previously inhabited by economically and racially privileged groups during apartheid. This group moved out of the inner-city during the 1990s and these areas are now home to a range of African migrants – from both within the country and across borders – who have claimed space in previously “forbidden ne cities”[26]. Today, many buildings and areas within these suburbs experience challenges associated with overcrowding, poor maintenance and problematic delivery in basic services.

A cluster-based random sampling technique was applied within each area. A detailed overview of the sampling strategy has been described previously [23]. A total of 195 households (40% of the total population surveyed) were interviewed in the informal settlement and 292 households (60% of the total population surveyed) in urban formal areas of the inner city.

### Measurements

The respondents were interviewed by trained fieldworkers using a structured questionnaire intended to collect information on socio-demographics and a range of issues including that of food security. To determine dietary diversity, the respondents were asked to recall the type of foods that they ate the day before [27]. This information was used to calculate the Dietary Diversity Score (DDS) by summing the number of food groups from which food had been consumed; the 9 food groups were (i) cereals, roots and tubers; (ii) vitamin A-rich vegetables and fruit; (iii) vegetables other than vitamin A-rich; (iv) fruit other than vitamin A-rich; (v) meat, poultry, and fish; (vi) eggs; (vii) legumes; (viii) dairy products; and (ix) fats or oils. Each group was counted only once. The lowest possible DDS therefore is zero and the highest possible score is 9. A DDS value of below 4 was considered low [18].

### Statistical analysis

In addition to descriptive analysis, statistical comparisons of means between groups were made with nonparametric ANOVA analysis. To assess the relationships between categorical variables, a chi-square analysis (Pearson’s method) was used. Statistical significance was set at p < 0.05.

Analyses were performed using JMP software package version 5.01 (SAS institute INC, Cary, NC, USA) and SPSS statistics 20 software package (IBM Corporation, NY, USA).

## Results

Table 1 shows the socio-demographic, migration and livelihood characteristics of the study population. Respondents from the informal settlement were older and have stayed for longer in Johannesburg. Only 9% of the total study sample has always lived in Johannesburg. Most (75.9%) of the respondents in the informal settlement were internal migrants, while in the formal settlement 49.7% were internal migrants and 44.2% were cross-border migrants. Cross-border migrants were the most likely to report that their food security has improved since moving to Johannesburg (data not shown). Both internal and cross border migrants in both informal and formal settlements did not receive agricultural produce or cash from “home” but remitted cash and goods, including food. Cross-border migrants remitted in greater numbers (60% versus 38% of internal migrants) and were more likely to remit food (30% versus 6%), most probably a function of the fact that many international migrants were from Zimbabwe where food shortages were acute at the time of the study.

**Table 1:** Summary table of socio-demographic, migration, health, environment, and livelihood characteristics of study population

	**Informal settlement *(n 195)***		**Formal settlement *(n 292)***		***P *value**^**1**^
	**Mean**	**SD**	**Mean**	**SD**	
Age of respondent (years)	35.6	12.2	31.3	10.1	<0.0001
Length of stay in Johannesburg (years)	5.4	3.7	3.8	4.4	<0.0001
Household size (number of people)	3.5	2.0	2.9	1.6	<0.0001
	**Informal settlement *(n 195)***		**Formal settlement *(n 292)***		***P *value**^**2**^
	%	[95% CI]	%	[95% CI]	
Female respondent	66.7	[59.8; 72.9]	50.0	[44.3; 55.7]	0.0003
Female headed household	35.0	[28.5; 41.8]	22.3	[17.8; 27.3]	0.0019
Migration status of respondent					
Internal migrant	75.9	[69.4; 81.4]	49.7	[43.9; 55.3]	<0.0001
Cross-border migrant	10.8	[7.1; 16.0]	44.2	[38.6; 49.9]	
Always resided in Johannesburg	13.3	[9.2; 18.8]	6.1	[3.8; 9.6]	
Satisfied with current residence	32.8	[26.6; 39.7]	67.8	[62.2; 72.9]	<0.0001
There are “more diseases where live now”	78.8	[72.7; 84.1]	57.8	[51.8; 63.0]	<0.0001
Running water inside household	35.5	[29.0; 42.3]	82.8	[78.1; 86.7]	<0.0001
Type of toilet					
Flush toilet inside household	35.2	[29.0; 42.3]	72.4	[66.8; 77.1]	<0.0001
Flush toilet outside household	25.3	[19.5; 31.7]	24.8	[20.0; 29.9]	
Communal toilet	10	[6.6; 15.4]	0	-	
Make use of the open bush	23.1	[17.7; 29.5]	0	-	
Other	6	[3.4; 10.5]	2.8	[1.3; 5.4]	
Fuel used for cooking					
Wood	7.7	[4.6; 12.4]	0.7	[0; 2.6]	<0.0001
Paraffin	75.4	[68.8; 80.9]	6.2	[3.9; 9.6]	
Gas	15.4	[10.9; 21.1]	0.7	[0; 2.6]	
Electricity	0	-	91.8	[88.0; 94.4]	
Other	1.5	[0.3; 4.6]	0.7	[0; 2.6]	
Fuel used for lighting					
Candles	79.7	[73.2; 84.6]	6.6	[4.1; 10.0]	<0.0001
Paraffin	20.3	[15.4; 26.7]	0	-	
Electricity	0	-	93.5	[90.0; 95.8]	
Refuse collection					
Burn rubbish	1.7	[0.3; 4.6]	1.1	[0.2; 3.1]	<0.0001
Dump rubbish outside yard	19.8	[14.9; 26.2]	1.8	[0.6; 4.0]	
Dump rubbish at dumpsite	22.0	[16.7; 28.4]	4.7	[2.5; 7.5]	
Rubbish is collected weekly	24.3	[18.6; 30.6]	92.0	[88.0; 94.4]	
Rubbish is collected irregularly	16.4	[11.8; 22.3]	0.4	[0; 2.0]	
Throw rubbish on the street	15.8	[11.4; 21.7]	0	-	
Tenure					
Own property	3.7	[1.6; 7.3]	6.9	[4.4; 10.4]	<0.0001
Constructed property	13.2	[9.2; 18.8]	0	-	
Rent property	4.7	[2.3; 8.6]	86.5	[81.8; 89.8]	
RDP or government housing	57.4	[50.4; 64.1]	1.0	[0.2; 3.1]	
Other	21.0	[15.8; 27.3]	5.5	[3.3; 8.7]	
Currently earning money (%)	41.1	[34.3; 48.0]	55.8	[50.0; 61.4]	0.0015
Social grants	43	[36.3; 50.1]	9.2	[6.4; 13.1]	<0.0001
Employment status	41.1	[34.3; 48.0]	57.7	[51.8; 63.0]	0.002
Experienced food shortages during previous 12 month	67.7	[60.8; 73.8]	55.5	[49.7; 61.0]	0.007
Food remittance	5.6	[3.0; 9.9]	30.1	[25.1; 35.6]	<0.0001

^**1**^ANOVA.

^2^chi-square analysis.

Table 1 further shows that residents from the informal settlement were less likely to have running water or a flush toilet inside their household, with nearly a quarter of the respondents having no access to toilet facilities (used the open bush). Rubbish collection was done weekly for most of the households in the formal settlements, while a significant number of households in the informal settlement dumped their rubbish either outside their yard, at the dumpsite or in the street, which poses a health risk. Most of the households (>90%) in the formal settlements used electricity for cooking and lighting, while in the informal settlement mostly paraffin was used for cooking and candles for lighting.

Residents of the informal settlement were more unlikely to be employed (59% versus 44%), and to have experienced food shortages during the previous year (67.7% versus 55%) than residents from the formal settlement.

Table 2 shows the proportion of respondents who consumed a food group at least once the previous day, food groups that were consumed by more than 50% of the respondents and the mean and 95% CI for the DDS for the total group, and per location and sex. No significant difference in mean DDS was observed between males and females, and consumption patterns of foods groups for both males and females were similar. The mean DDS for the total study sample was just over 4, while the mean DDS for the respondents in the informal settlement was 3.2. The mean DDS for respondents living in informal settlements was significantly lower than that for respondents living in formal settlements (ANOVA, p < 0.001). Significantly more respondents living in informal settlements consumed a diet of low diversity (68.1% versus 15.4%, p < 0.001). Respondents living in informal settlements consumed mostly cereals and, to a lesser extent, meat/poultry/fish, while respondents in the formal settlements consumed a more varied diet.

**Table 2:** Percentage of respondents who consumed these food groups the previous day, the mean dietary diversity score and the food groups consumed by more than 50% of the respondents, for the total study population and per settlement and sex

	**Total group**		**Settlement**				**Sex**			
			**Informal**		**Formal**		**Male**		**Female**	
N	487		195		292		210		274	
	%	[95% CI]	%	[95% CI]	%	[95% CI]	%	[95% CI]	%	[95% CI]
Cereals, roots and tubers	99.6	[98.4; 99.9]	99.5	[96.8; 99.9]	99.7	[97.8; 99.9]	99.0	[96.3; 99.9]	100.0	[98.3; 100]
Vitamin A-rich fruit & vegetables	28.2	[24.3; 32.2]	23.6	[18.1; 30.0]	31.2	[26.1; 36.7]	26.0	[20.2; 32.0]	29.7	[24.4; 35.2]
Vegetables other than vitamin A-rich	59.3	[54.7; 63.4]	43.5	[36.8; 50.6]	69.8	[64.3; 74.8]	58.5	[51.8; 65.0]	60.6	[54.6; 66.1]
Fruit other than vitamin A-rich	19.4	[16.0; 23.0]	12.6	[8.3; 17.7]	24.0	[19.4; 29.2]	17.9	[13.0; 23.3]	20.4	[16.0; 25.6]
Meat/poultry/fish	72.1	[67.9; 75.8]	50.8	[43.8; 57.7]	86.2	[81.8; 89.8]	72.9	[66.4; 78.4]	72.2	[66.6; 77.2]
Eggs	26.7	[22.9; 30.8]	16.2	[11.8; 22.2]	33.7	[28.4; 39.1]	28.5	[22.8; 35.0]	25.7	[20.7; 31.0]
Legumes	9.7	[7.3; 12.6]	11.0	[7.0; 15.9]	8.8	[6.1; 12.7]	7.4	[4.2; 11.5]	11.5	[8.0; 15.6]
Dairy products	33.0	[28.8; 37.1]	19.4	[14.5; 25.6]	42.0	[36.6; 47.8]	28.2	[22.4; 34.5]	36.7	[31.0; 42.3]
Fats/oils	65.8	[61.6; 70.0]	41.4	[34.8; 48.5]	82.0	[77.0; 85.8]	65.2	[58.5; 71.3]	66.7	[61.0; 72.1]
Dietary diversity score (DDS)										
Mean	4.1		3.2		4.8		4.0		4.2	
[95% CI]	[4.0; 4.3]		[3.0; 3.4]		[4.6; 5.0]		[3.8; 4.3]		[4.0; 4.4]	
Percentage DDS <4	36.8	[32.5; 41.2]	68.1	[61.1; 74.2]	15.4	[11.6; 20.1]	37.8	[31.3; 44.6]	35.7	[30.1; 41.6]
Food groups consumed by more than 50% of the respondents	Cereals		Cereals		Cereals		Cereals		Cereals	
	Vegetables*		Meat, poultry, fish		Vegetables*		Vegetables*		Vegetables*	
	Meat, poultry, fish				Meat, poultry, fish		Meat, poultry, fish		Meat, poultry, fish	
	Fats and oils				Fats and oils		Fats and oils		Fats and oils	

All values are given as a percentage and [95% CI], except for the dietary diversity score.

The sex was missing for 3 respondents.

DDS values missing for 17 respondents because of incomplete data for the nine food groups.

Mean DDS ANOVA: informal versus formal p < 0.001.

Males versus females p = 0.229.

Percentage DDS <4 chi-square: informal versus formal p < 0.001.

Males versus females p = 0.641.

* other than vitamin A-rich.

The percentage of households reportedly affected by HIV was similar for the informal (4.8%) and formal (5.2%) settings. Although not statistically significant, fewer respondents from HIV-affected households consumed a diet of low variety (DDS < 4; 20.8% versus 37.5%; P = 0.099). For respondents from HIV-affected households, consumption of fats and oils (87.5% versus 64.7%; p = 0.022) and vegetables other than vitamin A-rich (79.2% versus 58.6%, p = 0.045) was higher than those from non-HIV affected households.

The mean DDS for respondents from households who experienced periods of food shortages during the previous 12 months was significantly lower than those from food secure households (4.00 ± 1.6 versus 4.36 ± 1.7; p = 0.026). Those who had experienced periods of food shortages consumed less from the fruit other than vitamin A-rich (14.1 versus 27.7%; p < 0.001), meat/ poultry/fish (68.4% versus 77.8%; p = 0.025) and eggs (23.4% versus 31.7%; p = 0.045) groups, and more from the vegetables other than vitamin-A rich group (63.9% versus 52.1%; p = 0.010).

Based on the frequency distribution of the DDS for the total study sample, the respondents were grouped into five more-or-less equal (20%) groups. These five groups were defined as two or fewer food groups (n = 85; 18%); three food groups (n = 88; 19%), four food groups (n = 114; 24%) five food groups (n = 90; 19%) and six or more food groups (n = 93; 20%). The percentage of respondents consuming different food groups and the food groups consumed by more than 50% of the respondents per DDS quintile is given in Table 3. The distribution of respondents across the five DDS quintiles according to sex and type of settlement is also shown in Table 3. Males and females showed the same distribution pattern over the five quintiles. Two-thirds of the respondents from informal settlements fell in the lowest two quintiles, versus only 15.4% of the respondents living in formal settlements, highlighting the nutritional vulnerability of the informal residents.

**Table 3:** The percentage of respondents consuming different food groups, food groups consumed by more than 50% of respondents per DDS quintile for the total study population, and the frequency distribution of respondents over the quintiles according to type of settlement and sex

		**Quintile 1**	**Quintile 2**	**Quintile 3**	**Quintile 4**	**Quintile 5**
		**(1–2 food groups)**	**(3 food groups)**	**(4 food groups)**	**(5 food groups)**	**(6 or more food groups)**
N		85	88	114	90	93
% respondents		18	19	24	19	20
Cereals, roots and tubers		97.6	100	100	100	100
Vitamin A-rich fruit & vegetables		8.2	19.3	13.2	42.2	60.2
Vegetables other than vitamin A-rich		25.9	31.8	62.3	84.4	89.2
Fruit other than vitamin A-rich		2.4	5.7	8.8	23.3	59.1
Meat/poultry/fish		35.3	59.1	80.7	85.6	95.7
Eggs		0.0	19.3	17.5	34.4	61.3
Legumes		3.5	8.0	7.0	5.6	23.7
Dairy products		3.5	10.2	31.6	35.6	79.6
Fats/oils		4.7	46.6	78.9	88.9	100.0
Food groups consumed by > 50% of respondents		Cereals, roots & tubers	Cereals, roots & tubers	Cereals, roots & tubers	Cereals, roots & tubers	Cereals, roots & tubers
			Meat/poultry/fish	Meat/poultry/fish	Meat/poultry/fish	Meat/poultry/fish
				Vegetables*	Vegetables*	Vegetables*
				Fats/oils	Fats/oils	Fats/oils
						Vit A-rich fruit & vegetables
						Fruit*
						Eggs
						Dairy products
Settlement	Informal	37.2	30.9	14.7	9.9	7.3
	Formal	5.0	10.4	30.8	25.4	28.3
Sex	Males	17.9	19.9	25.4	19.9	16.9
	Females	17.7	18.0	23.3	18.8	22.2

* other than vitamin A-rich.

## Discussion and conclusions

This study showed that dietary diversity was low for the majority of this urban study population, with residents of informal settlements having the lowest dietary diversity. Respondents from households that experienced food shortages during the previous 12 months consumed a diet of lower diversity, suggesting that they were nutritionally more vulnerable. Although the dietary diversity score is based on a single 24-hr recall and reflects current status, a relation between dietary diversity and longer term indicators such as food shortages during the previous 12 months and household food insecurity has previously been reported [28]. In a recent study that focused on a similar environment, the authors concluded that food insecurity was pervasive in a poverty-stricken community living informally with the result that caregivers changed their food consumption patterns to cope, resulting in compromised nutrition [29]. This was reiterated in a national study that emphasized that dietary diversity was particularly low in urban informal areas across the country [22].

The importance of consuming a variety of foods is captured in the South African food-based dietary guideline “Enjoy a variety of foods”. These guidelines may remain “academic” in nature as the poor, in many instances, lack the resources to obtain a variety of foods. Previous studies have shown lower dietary diversity in the lower living standard measure (LSM) groups in South Africa [22; 28], reflecting poor people’s ability to access a large variety of foods. One particular study in South Africa reported similar seasonal patterns for months of inadequate food provision and shortage of money, highlighting the importance of household income for food security [28]. Temple and Steyn argued that most South Africans cannot afford a healthy diet, as it costs on average 69% more than the unhealthy food choices they make presently [30]. As a result of the cost of healthy foods, lower socio-economic groups drift towards poor quality, energy-dense but cheap foods [31]. Vorster et al. argued that the reliance on available and affordable staple foods and energy-dense but nutrient-poor foods, snacks and beverages contributes towards the increased vulnerability to the nutrition transition in Africa [32]. Other studies have shown that lower calorie, nutrient-dense, less-processed foods such as fruits and vegetables generally do cost more, and that cost is a barrier to the urban poor, in both a South African and broader context [33]–[35]. Less healthy foods also tend to cost less, which was confirmed by a recent study of food prices in fourteen towns in the Western Cape in South Africa that compared the prices of six commonly consumed foods with healthier versions of those foods [36].

Health promotion strategies to improve dietary diversity (such as the food-based dietary guidelines) are likely to achieve only limited success in a context of inadequate food affordability. Recommendations therefore need to be carefully crafted, especially when most people in the target population have a low income. Overcoming this barrier will require a range of responses at different scales including attempts to lower food prices, which by implication requires government intervention in accessibility through taxation and subsidies. Another strategy is to ensure healthy food choices in nutrition programmes. For example, providing food aid to the elderly at a care centre was shown to result in a significant improvement in dietary diversity [37]. Besides economic factors, other factors such as taste, convenience and poor physical access to affordable foods may also lead to the selection of an unhealthy diet. Dietary diversity scores should be interpreted cautiously. For example, frequent consumers of fast foods in South Africa were shown to have a higher dietary diversity score [38]. It is important to note that the dietary guideline “Enjoy a variety of foods” does not promote an increased consumption of fast foods [39].

Within this context, a key question is how can policy respond? While there are several barriers between the general population and a healthier diet, cost is probably the most important factor for South Africans in gaining access to healthier food. Thus the challenge is how to promote access to diverse, quality foods that are financially accessible in these communities. An important option is to assist the development of local markets in close proximity to informal areas, which will entail supporting vendors to access such foods directly from local producers to ensure cost controls. However, all these challenges and potential solutions highlight challenges around inter-sectoral collaboration within local government structures. Ensuring nutrition security in urban informal settlements requires the alignment and engagement between those responsible for housing, informal settlement upgrading, environmental health, transport, social development and a range of other services. As such, collaboration is a challenge in itself, recognizing the importance of urban nutrition security must become a galvanizing factor to encourage such response.

Using the measurement of dietary diversity as a proxy measure for nutritional quality of the diet, many of those surveyed consumed a nutritionally inadequate diet, with households residing in the urban informal settlements being more nutritionally vulnerable. Households residing informally also had poor access to basic services, such as clean water, electricity and healthcare, all of which have been demonstrated to play a role in producing vulnerability to disease [40,41]. Urban poverty, particularly in urban informal areas, creates the social and environmental context that promotes nutrition insecurity underscoring the fact that undernutrition is taking on an increasingly urban character. Achieving nutrition security requires that development policies, strategies and plans include specific nutrition objectives and considerations. The challenge for policymakers and analysts concerned with urban development and the dimension of achieving nutrition security is to understand the links between the availability of food, accessing this food, consumption and nutritional status. This is particularly challenging in South Africa given the range of issues affecting nutritional outcomes – but a challenge that needs to be addressed for the successful development of urban areas.

## Competing interests

The authors declare that they have no competing interest.

## Authors’ contributions

SD undertook the main drafting of this manuscript and provided oversight for the study. JV and LN led the research team in Johannesburg, undertook the primary analysis and drafted the manuscript. MF undertook the analysis around dietary diversity and drafted the manuscript. All authors read and approved the final manuscript.

## Pre-publication history

The pre-publication history for this paper can be accessed here:

http://www.biomedcentral.com/1471-2458/13/911/prepub
